# Data set for Tifinagh handwriting character recognition

**DOI:** 10.1016/j.dib.2015.04.008

**Published:** 2015-04-23

**Authors:** Omar Bencharef, Younes Chihab, Nouredine Mousaid, Mustapha Oujaoura

**Affiliations:** a Higher School of Technology-Essaouira, Cadi Ayyad University, Marrakech, Morocco; b FP Safi, Cadi Ayyad University, Marrakech, Morocco; c Faculty of Science and Technology, Sultan Moulay Slimane University, Beni Mellal, Morocco

## Abstract

The Tifinagh alphabet-IRCAM is the official alphabet of the Amazigh language widely used in North Africa [1]. It includes thirty-one basic letter and two letters each composed of a base letter followed by the sign of labialization. Normalized only in 2003 (Unicode) [2], ICRAM-Tifinagh is a young character repertoire. Which needs more work on all levels. In this context we propose a data set for handwritten Tifinagh characters composed of 1376 image; 43 Image For Each character. The dataset can be used to train a Tifinagh character recognition system, or to extract the meaning characteristics of each character.

## 1 Specifications table

**Table:** 

Subject area	Computer science

More specific subject area	Image processing, character recognition
Type of data	Image
How data was acquired	Hand writing and scanner
Data format	Jpg image
Experimental factors	We ask 30 students to write in each cell of a table all Tifinagh characters, we use an Epson 10000XL to data scan, and we add 13 more features to take on consideration horizontal and vertical inclination
Experimental features	1376 Image with a size of 30⁎30px (43 images/character)
Data source location	Essaouira, Morocco
Data accessibility	Within this article

## 2 Value of the data

• The Amazigh language is considered as official language only in 2003. Therefore, The integration of the Tifinagh alphabet in new information technologies and communication (ICT) and engage in research in this field has become a major necessity [1], [2].
• The Amazigh language is spoken by about 30 million people in North Africa (the oasis of Siwa in Egypt, Morocco through Libya, Tunisia, Algeria, Niger, Mali, Burkina Faso and Mauritania) [3], [4].
• Due to the diversity of hand writing characters, there are two big approaches in this field and both need a dataset to be executed: the first one is based on complex classifiers like Artificial Neural Network or Support Vector Machine; those classifiers need a dataset to be trained to classify characters[5]. The other approaches also need a dataset this time to find a normalization of each character.
• The data set is very useful to train classification system for Tifinagh hand writing, that remain an active area of research.
• The dataset is the first free and on line dataset for handwriting Tifinagh character without formalities.

## 3 Experimental design, materials and methods

**Fig. 1:**
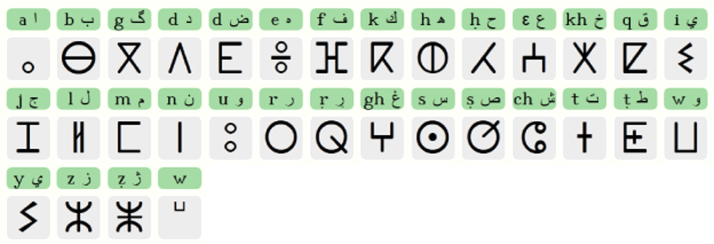
Elementary IRCAM Tifinagh characters.

We ask 30 people (17 male and 13 female) to write the 32 Tifinagh (Fig. 1) characters on one page, and we add 13 more features to take on consideration horizontal and vertical inclination. The pages where scanned using the Epson 10000XL.

**Fig. 2:**
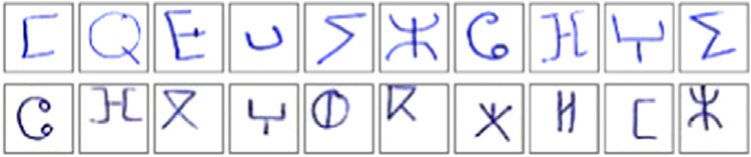
Example of handwriting Tifinagh character from the proposed data base.
• The sub-image are named using Latin character mentioned in Fig. 1 for each character followed by number from 1 to 30( a1,a2…a43 ). For character with sub point we use a double character (hh or zz). For epsilon we use a double A(aa1,aa2…aa43).

The extraction steps were:

• We use the horizontal histogram to correct the inclination of every page [6].
• Using connected components algorithm we detect the center of each character [7].
• We extract 31 sub-images of 30×30px that contain the characters (Fig. 2).

To automatically explore the dataset or to extract features from the whole dataset we propose the following Matlab code:

**Table:** 

function x=base_generation()
//Read all jpg image from folder ‘data_set’
fileFolder = fullfile(‘data_set’);
dirOutput = dir(fullfile(fileFolder,’⁎.jpg’))
fileNames = {dirOutput.name}׳
numFrames = numel(fileNames)
cd ‘data_set׳
p = imread(fileNames{2});
//We read and converts to gray level the first image then we call the //feature extraction process
d=imread(p);
d=double(d)/255;
y=rgb2gray(d);
t =zmoment(y,11); // Call the feature extraction
b=t; //we add the ‘t’ to the data matrix
// We repeat the same treatment for the rest of the data set
for i=2:1240
p=m{i};
d=imread(p);
d=double(d)/255;
y=rgb2gray(d);
**t=zmoment(y,11);** // Call the feature extraction function(Zernike for // example)
b=[b;t];
End
x=b
