# Supplementary figures and images for: Data set for Tifinagh handwriting character recognition (part 2 of 2)
Source: Data Brief. 2015 Apr 23;4:11–3. doi: 10.1016/j.dib.2015.04.008 (PMC4510372; doi:10.1016/j.dib.2015.04.008)

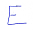

Supplement: Supplementary file 1 — Supplementary data [file mmc1.zip › 03/dd3.png]

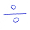

Supplement: Supplementary file 1 — Supplementary data [file mmc1.zip › 03/e3.png]

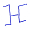

Supplement: Supplementary file 1 — Supplementary data [file mmc1.zip › 03/f3.png]

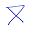

Supplement: Supplementary file 1 — Supplementary data [file mmc1.zip › 03/g3.png]

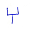

Supplement: Supplementary file 1 — Supplementary data [file mmc1.zip › 03/gh3.png]

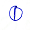

Supplement: Supplementary file 1 — Supplementary data [file mmc1.zip › 03/h3.png]

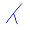

Supplement: Supplementary file 1 — Supplementary data [file mmc1.zip › 03/hh3.png]

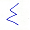

Supplement: Supplementary file 1 — Supplementary data [file mmc1.zip › 03/i3.png]

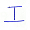

Supplement: Supplementary file 1 — Supplementary data [file mmc1.zip › 03/j3.png]

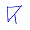

Supplement: Supplementary file 1 — Supplementary data [file mmc1.zip › 03/k3.png]

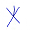

Supplement: Supplementary file 1 — Supplementary data [file mmc1.zip › 03/kh3.png]

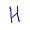

Supplement: Supplementary file 1 — Supplementary data [file mmc1.zip › 03/l3.png]

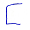

Supplement: Supplementary file 1 — Supplementary data [file mmc1.zip › 03/m3.png]

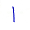

Supplement: Supplementary file 1 — Supplementary data [file mmc1.zip › 03/n3.png]

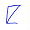

Supplement: Supplementary file 1 — Supplementary data [file mmc1.zip › 03/q3.png]

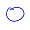

Supplement: Supplementary file 1 — Supplementary data [file mmc1.zip › 03/r3.png]

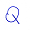

Supplement: Supplementary file 1 — Supplementary data [file mmc1.zip › 03/rr3.png]

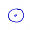

Supplement: Supplementary file 1 — Supplementary data [file mmc1.zip › 03/s3.png]

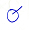

Supplement: Supplementary file 1 — Supplementary data [file mmc1.zip › 03/ss3.png]

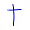

Supplement: Supplementary file 1 — Supplementary data [file mmc1.zip › 03/t3.png]

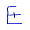

Supplement: Supplementary file 1 — Supplementary data [file mmc1.zip › 03/tt3.png]

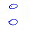

Supplement: Supplementary file 1 — Supplementary data [file mmc1.zip › 03/u3.png]

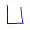

Supplement: Supplementary file 1 — Supplementary data [file mmc1.zip › 03/w3.png]

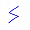

Supplement: Supplementary file 1 — Supplementary data [file mmc1.zip › 03/y3.png]

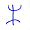

Supplement: Supplementary file 1 — Supplementary data [file mmc1.zip › 03/z3.png]

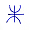

Supplement: Supplementary file 1 — Supplementary data [file mmc1.zip › 03/zz3.png]

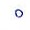

Supplement: Supplementary file 1 — Supplementary data [file mmc1.zip › 04/A4.jpg]

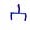

Supplement: Supplementary file 1 — Supplementary data [file mmc1.zip › 04/AA4.jpg]

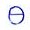

Supplement: Supplementary file 1 — Supplementary data [file mmc1.zip › 04/B4.jpg]

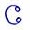

Supplement: Supplementary file 1 — Supplementary data [file mmc1.zip › 04/CH4.jpg]

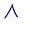

Supplement: Supplementary file 1 — Supplementary data [file mmc1.zip › 04/D4.jpg]

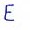

Supplement: Supplementary file 1 — Supplementary data [file mmc1.zip › 04/DD4.jpg]

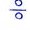

Supplement: Supplementary file 1 — Supplementary data [file mmc1.zip › 04/E4.jpg]

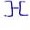

Supplement: Supplementary file 1 — Supplementary data [file mmc1.zip › 04/F4.jpg]

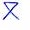

Supplement: Supplementary file 1 — Supplementary data [file mmc1.zip › 04/G4.jpg]

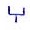

Supplement: Supplementary file 1 — Supplementary data [file mmc1.zip › 04/GH4.jpg]

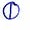

Supplement: Supplementary file 1 — Supplementary data [file mmc1.zip › 04/H4.jpg]

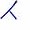

Supplement: Supplementary file 1 — Supplementary data [file mmc1.zip › 04/HH4.jpg]

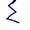

Supplement: Supplementary file 1 — Supplementary data [file mmc1.zip › 04/I4.jpg]

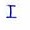

Supplement: Supplementary file 1 — Supplementary data [file mmc1.zip › 04/J4.jpg]

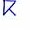

Supplement: Supplementary file 1 — Supplementary data [file mmc1.zip › 04/K4.jpg]

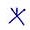

Supplement: Supplementary file 1 — Supplementary data [file mmc1.zip › 04/KH4.jpg]

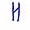

Supplement: Supplementary file 1 — Supplementary data [file mmc1.zip › 04/L4.jpg]

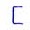

Supplement: Supplementary file 1 — Supplementary data [file mmc1.zip › 04/M4.jpg]

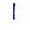

Supplement: Supplementary file 1 — Supplementary data [file mmc1.zip › 04/N4.jpg]

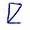

Supplement: Supplementary file 1 — Supplementary data [file mmc1.zip › 04/Q4.jpg]

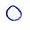

Supplement: Supplementary file 1 — Supplementary data [file mmc1.zip › 04/R4.jpg]

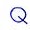

Supplement: Supplementary file 1 — Supplementary data [file mmc1.zip › 04/RR4.jpg]

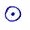

Supplement: Supplementary file 1 — Supplementary data [file mmc1.zip › 04/S4.jpg]

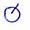

Supplement: Supplementary file 1 — Supplementary data [file mmc1.zip › 04/SS4.jpg]

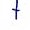

Supplement: Supplementary file 1 — Supplementary data [file mmc1.zip › 04/T4.jpg]

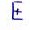

Supplement: Supplementary file 1 — Supplementary data [file mmc1.zip › 04/TT4.jpg]

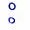

Supplement: Supplementary file 1 — Supplementary data [file mmc1.zip › 04/U4.jpg]

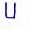

Supplement: Supplementary file 1 — Supplementary data [file mmc1.zip › 04/W4.jpg]

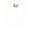

Supplement: Supplementary file 1 — Supplementary data [file mmc1.zip › 04/WW4.jpg]

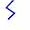

Supplement: Supplementary file 1 — Supplementary data [file mmc1.zip › 04/Y4.jpg]

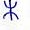

Supplement: Supplementary file 1 — Supplementary data [file mmc1.zip › 04/Z4.jpg]

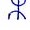

Supplement: Supplementary file 1 — Supplementary data [file mmc1.zip › 04/ZZ4.jpg]

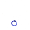

Supplement: Supplementary file 1 — Supplementary data [file mmc1.zip › 05/a5.png]

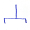

Supplement: Supplementary file 1 — Supplementary data [file mmc1.zip › 05/aa5.png]

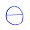

Supplement: Supplementary file 1 — Supplementary data [file mmc1.zip › 05/b5.png]

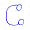

Supplement: Supplementary file 1 — Supplementary data [file mmc1.zip › 05/ch5.png]

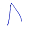

Supplement: Supplementary file 1 — Supplementary data [file mmc1.zip › 05/d5.png]

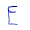

Supplement: Supplementary file 1 — Supplementary data [file mmc1.zip › 05/dd5.png]

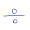

Supplement: Supplementary file 1 — Supplementary data [file mmc1.zip › 05/e5.png]

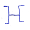

Supplement: Supplementary file 1 — Supplementary data [file mmc1.zip › 05/f5.png]

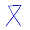

Supplement: Supplementary file 1 — Supplementary data [file mmc1.zip › 05/g5.png]

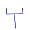

Supplement: Supplementary file 1 — Supplementary data [file mmc1.zip › 05/gh5.png]

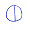

Supplement: Supplementary file 1 — Supplementary data [file mmc1.zip › 05/h5.png]

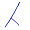

Supplement: Supplementary file 1 — Supplementary data [file mmc1.zip › 05/hh5.png]

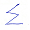

Supplement: Supplementary file 1 — Supplementary data [file mmc1.zip › 05/i5.png]

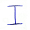

Supplement: Supplementary file 1 — Supplementary data [file mmc1.zip › 05/j5.png]

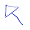

Supplement: Supplementary file 1 — Supplementary data [file mmc1.zip › 05/k5.png]

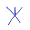

Supplement: Supplementary file 1 — Supplementary data [file mmc1.zip › 05/kh5.png]

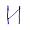

Supplement: Supplementary file 1 — Supplementary data [file mmc1.zip › 05/l5.png]

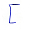

Supplement: Supplementary file 1 — Supplementary data [file mmc1.zip › 05/m5.png]

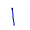

Supplement: Supplementary file 1 — Supplementary data [file mmc1.zip › 05/n5.png]

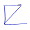

Supplement: Supplementary file 1 — Supplementary data [file mmc1.zip › 05/q5.png]

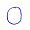

Supplement: Supplementary file 1 — Supplementary data [file mmc1.zip › 05/r5.png]

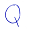

Supplement: Supplementary file 1 — Supplementary data [file mmc1.zip › 05/rr5.png]

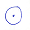

Supplement: Supplementary file 1 — Supplementary data [file mmc1.zip › 05/s5.png]

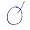

Supplement: Supplementary file 1 — Supplementary data [file mmc1.zip › 05/ss5.png]

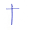

Supplement: Supplementary file 1 — Supplementary data [file mmc1.zip › 05/t5.png]

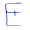

Supplement: Supplementary file 1 — Supplementary data [file mmc1.zip › 05/tt5.png]

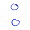

Supplement: Supplementary file 1 — Supplementary data [file mmc1.zip › 05/u5.png]

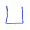

Supplement: Supplementary file 1 — Supplementary data [file mmc1.zip › 05/w5.png]

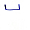

Supplement: Supplementary file 1 — Supplementary data [file mmc1.zip › 05/ww5.png]

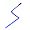

Supplement: Supplementary file 1 — Supplementary data [file mmc1.zip › 05/y5.png]

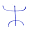

Supplement: Supplementary file 1 — Supplementary data [file mmc1.zip › 05/z5.png]

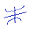

Supplement: Supplementary file 1 — Supplementary data [file mmc1.zip › 05/zz5.png]

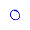

Supplement: Supplementary file 1 — Supplementary data [file mmc1.zip › 06/a6.png]

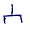

Supplement: Supplementary file 1 — Supplementary data [file mmc1.zip › 06/aa6.png]

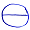

Supplement: Supplementary file 1 — Supplementary data [file mmc1.zip › 06/b6.png]

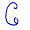

Supplement: Supplementary file 1 — Supplementary data [file mmc1.zip › 06/ch6.png]

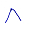

Supplement: Supplementary file 1 — Supplementary data [file mmc1.zip › 06/d6.png]

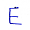

Supplement: Supplementary file 1 — Supplementary data [file mmc1.zip › 06/dd6.png]

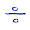

Supplement: Supplementary file 1 — Supplementary data [file mmc1.zip › 06/e6.png]

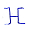

Supplement: Supplementary file 1 — Supplementary data [file mmc1.zip › 06/f6.png]
