# Supplementary figures and images for: Data set for Tifinagh handwriting character recognition (part 1 of 2)
Source: Data Brief. 2015 Apr 23;4:11–3. doi: 10.1016/j.dib.2015.04.008 (PMC4510372; doi:10.1016/j.dib.2015.04.008)

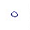

Supplement: Supplementary file 1 — Supplementary data [file mmc1.zip › 00/a.png]

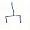

Supplement: Supplementary file 1 — Supplementary data [file mmc1.zip › 00/aa.png]

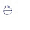

Supplement: Supplementary file 1 — Supplementary data [file mmc1.zip › 00/b.png]

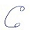

Supplement: Supplementary file 1 — Supplementary data [file mmc1.zip › 00/ch.png]

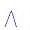

Supplement: Supplementary file 1 — Supplementary data [file mmc1.zip › 00/d1.png]

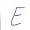

Supplement: Supplementary file 1 — Supplementary data [file mmc1.zip › 00/dd.png]

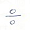

Supplement: Supplementary file 1 — Supplementary data [file mmc1.zip › 00/e.png]

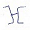

Supplement: Supplementary file 1 — Supplementary data [file mmc1.zip › 00/f.png]

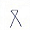

Supplement: Supplementary file 1 — Supplementary data [file mmc1.zip › 00/g.png]

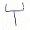

Supplement: Supplementary file 1 — Supplementary data [file mmc1.zip › 00/gh.png]

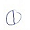

Supplement: Supplementary file 1 — Supplementary data [file mmc1.zip › 00/h.png]

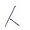

Supplement: Supplementary file 1 — Supplementary data [file mmc1.zip › 00/hh.png]

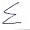

Supplement: Supplementary file 1 — Supplementary data [file mmc1.zip › 00/i.png]

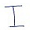

Supplement: Supplementary file 1 — Supplementary data [file mmc1.zip › 00/j.png]

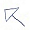

Supplement: Supplementary file 1 — Supplementary data [file mmc1.zip › 00/k.png]

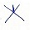

Supplement: Supplementary file 1 — Supplementary data [file mmc1.zip › 00/kh.png]

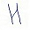

Supplement: Supplementary file 1 — Supplementary data [file mmc1.zip › 00/l.png]

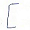

Supplement: Supplementary file 1 — Supplementary data [file mmc1.zip › 00/m.png]

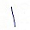

Supplement: Supplementary file 1 — Supplementary data [file mmc1.zip › 00/n.png]

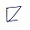

Supplement: Supplementary file 1 — Supplementary data [file mmc1.zip › 00/q.png]

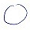

Supplement: Supplementary file 1 — Supplementary data [file mmc1.zip › 00/r.png]

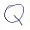

Supplement: Supplementary file 1 — Supplementary data [file mmc1.zip › 00/rr.png]

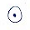

Supplement: Supplementary file 1 — Supplementary data [file mmc1.zip › 00/s.png]

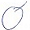

Supplement: Supplementary file 1 — Supplementary data [file mmc1.zip › 00/ss.png]

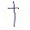

Supplement: Supplementary file 1 — Supplementary data [file mmc1.zip › 00/t.png]

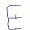

Supplement: Supplementary file 1 — Supplementary data [file mmc1.zip › 00/tt.png]

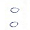

Supplement: Supplementary file 1 — Supplementary data [file mmc1.zip › 00/u.png]

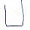

Supplement: Supplementary file 1 — Supplementary data [file mmc1.zip › 00/w.png]

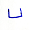

Supplement: Supplementary file 1 — Supplementary data [file mmc1.zip › 00/ww.png]

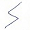

Supplement: Supplementary file 1 — Supplementary data [file mmc1.zip › 00/y.png]

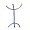

Supplement: Supplementary file 1 — Supplementary data [file mmc1.zip › 00/z.png]

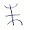

Supplement: Supplementary file 1 — Supplementary data [file mmc1.zip › 00/zz.png]

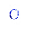

Supplement: Supplementary file 1 — Supplementary data [file mmc1.zip › 01/a1.png]

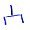

Supplement: Supplementary file 1 — Supplementary data [file mmc1.zip › 01/aa1.png]

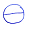

Supplement: Supplementary file 1 — Supplementary data [file mmc1.zip › 01/b1.png]

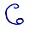

Supplement: Supplementary file 1 — Supplementary data [file mmc1.zip › 01/ch1.png]

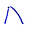

Supplement: Supplementary file 1 — Supplementary data [file mmc1.zip › 01/d1.png]

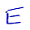

Supplement: Supplementary file 1 — Supplementary data [file mmc1.zip › 01/dd1.png]

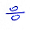

Supplement: Supplementary file 1 — Supplementary data [file mmc1.zip › 01/e1.png]

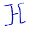

Supplement: Supplementary file 1 — Supplementary data [file mmc1.zip › 01/f1.png]

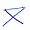

Supplement: Supplementary file 1 — Supplementary data [file mmc1.zip › 01/g1.png]

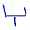

Supplement: Supplementary file 1 — Supplementary data [file mmc1.zip › 01/gh1.png]

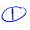

Supplement: Supplementary file 1 — Supplementary data [file mmc1.zip › 01/h1.png]

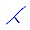

Supplement: Supplementary file 1 — Supplementary data [file mmc1.zip › 01/hh1.png]

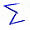

Supplement: Supplementary file 1 — Supplementary data [file mmc1.zip › 01/i1.png]

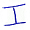

Supplement: Supplementary file 1 — Supplementary data [file mmc1.zip › 01/j1.png]

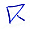

Supplement: Supplementary file 1 — Supplementary data [file mmc1.zip › 01/k1.png]

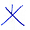

Supplement: Supplementary file 1 — Supplementary data [file mmc1.zip › 01/kh1.png]

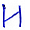

Supplement: Supplementary file 1 — Supplementary data [file mmc1.zip › 01/l1.png]

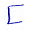

Supplement: Supplementary file 1 — Supplementary data [file mmc1.zip › 01/m1.png]

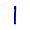

Supplement: Supplementary file 1 — Supplementary data [file mmc1.zip › 01/n1.png]

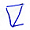

Supplement: Supplementary file 1 — Supplementary data [file mmc1.zip › 01/q1.png]

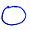

Supplement: Supplementary file 1 — Supplementary data [file mmc1.zip › 01/r1.png]

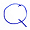

Supplement: Supplementary file 1 — Supplementary data [file mmc1.zip › 01/rr1.png]

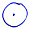

Supplement: Supplementary file 1 — Supplementary data [file mmc1.zip › 01/s1.png]

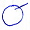

Supplement: Supplementary file 1 — Supplementary data [file mmc1.zip › 01/ss1.png]

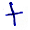

Supplement: Supplementary file 1 — Supplementary data [file mmc1.zip › 01/t1.png]

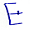

Supplement: Supplementary file 1 — Supplementary data [file mmc1.zip › 01/tt1.png]

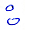

Supplement: Supplementary file 1 — Supplementary data [file mmc1.zip › 01/u1.png]

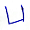

Supplement: Supplementary file 1 — Supplementary data [file mmc1.zip › 01/w1.png]

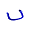

Supplement: Supplementary file 1 — Supplementary data [file mmc1.zip › 01/ww1.png]

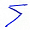

Supplement: Supplementary file 1 — Supplementary data [file mmc1.zip › 01/y1.png]

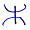

Supplement: Supplementary file 1 — Supplementary data [file mmc1.zip › 01/z1.png]

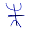

Supplement: Supplementary file 1 — Supplementary data [file mmc1.zip › 01/ZZ1.png]

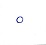

Supplement: Supplementary file 1 — Supplementary data [file mmc1.zip › 02/a2.jpg]

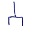

Supplement: Supplementary file 1 — Supplementary data [file mmc1.zip › 02/aa2.jpg]

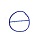

Supplement: Supplementary file 1 — Supplementary data [file mmc1.zip › 02/b2.jpg]

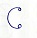

Supplement: Supplementary file 1 — Supplementary data [file mmc1.zip › 02/ch2.jpg]

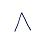

Supplement: Supplementary file 1 — Supplementary data [file mmc1.zip › 02/d2.jpg]

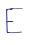

Supplement: Supplementary file 1 — Supplementary data [file mmc1.zip › 02/dd2.jpg]

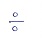

Supplement: Supplementary file 1 — Supplementary data [file mmc1.zip › 02/e2.jpg]

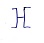

Supplement: Supplementary file 1 — Supplementary data [file mmc1.zip › 02/f2.jpg]

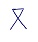

Supplement: Supplementary file 1 — Supplementary data [file mmc1.zip › 02/g2.jpg]

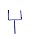

Supplement: Supplementary file 1 — Supplementary data [file mmc1.zip › 02/gh2.jpg]

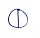

Supplement: Supplementary file 1 — Supplementary data [file mmc1.zip › 02/h2.jpg]

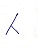

Supplement: Supplementary file 1 — Supplementary data [file mmc1.zip › 02/hh2.jpg]

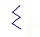

Supplement: Supplementary file 1 — Supplementary data [file mmc1.zip › 02/i2.jpg]

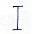

Supplement: Supplementary file 1 — Supplementary data [file mmc1.zip › 02/j2.jpg]

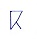

Supplement: Supplementary file 1 — Supplementary data [file mmc1.zip › 02/K2.jpg]

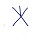

Supplement: Supplementary file 1 — Supplementary data [file mmc1.zip › 02/kh2.jpg]

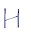

Supplement: Supplementary file 1 — Supplementary data [file mmc1.zip › 02/l2.jpg]

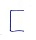

Supplement: Supplementary file 1 — Supplementary data [file mmc1.zip › 02/m2.jpg]

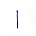

Supplement: Supplementary file 1 — Supplementary data [file mmc1.zip › 02/n2.jpg]

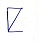

Supplement: Supplementary file 1 — Supplementary data [file mmc1.zip › 02/q2.jpg]

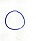

Supplement: Supplementary file 1 — Supplementary data [file mmc1.zip › 02/r2.jpg]

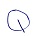

Supplement: Supplementary file 1 — Supplementary data [file mmc1.zip › 02/rr2.jpg]

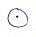

Supplement: Supplementary file 1 — Supplementary data [file mmc1.zip › 02/s2.jpg]

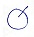

Supplement: Supplementary file 1 — Supplementary data [file mmc1.zip › 02/ss2.jpg]

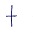

Supplement: Supplementary file 1 — Supplementary data [file mmc1.zip › 02/t2.jpg]

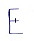

Supplement: Supplementary file 1 — Supplementary data [file mmc1.zip › 02/tt2.jpg]

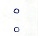

Supplement: Supplementary file 1 — Supplementary data [file mmc1.zip › 02/u2.jpg]

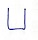

Supplement: Supplementary file 1 — Supplementary data [file mmc1.zip › 02/w2.jpg]

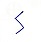

Supplement: Supplementary file 1 — Supplementary data [file mmc1.zip › 02/y2.jpg]

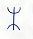

Supplement: Supplementary file 1 — Supplementary data [file mmc1.zip › 02/z2.jpg]

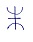

Supplement: Supplementary file 1 — Supplementary data [file mmc1.zip › 02/zz2.jpg]

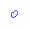

Supplement: Supplementary file 1 — Supplementary data [file mmc1.zip › 03/a3.png]

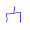

Supplement: Supplementary file 1 — Supplementary data [file mmc1.zip › 03/aa3.png]

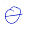

Supplement: Supplementary file 1 — Supplementary data [file mmc1.zip › 03/b3.png]

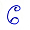

Supplement: Supplementary file 1 — Supplementary data [file mmc1.zip › 03/ch3.png]

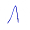

Supplement: Supplementary file 1 — Supplementary data [file mmc1.zip › 03/d3.png]
